# Effect of crossbreeding on growth performance, meat quality, and the economics of production of the pure and reciprocal crosses between the Sasso and Wassachie chickens

**DOI:** 10.1016/j.psj.2024.103823

**Published:** 2024-05-17

**Authors:** Joshua T. Dzungwe, Benjamin Adjei-Mensah, Christophe A.A.M. Chrysostome, Koffi Tozo

**Affiliations:** ⁎ Regional Center of Excellence for Poultry Science (CERSA), University of Lome, Lome, Togo; † Department of Animal Production, University of Abomey-Cavali, Benin, Africa; ‡ Department of Animal Breeding and Physiology, Joseph Sarwuan Tarka University, Makurdi, Nigeria

**Keywords:** growth-trait, meat-quality, drip-loss, cooking-loss, sensory-evaluation

## Abstract

The interplay between genetics and economics is important in understanding how crossbreeding can be harnessed to optimize sustainable poultry production, meat quality, and economic viability. This study was conducted to investigate the effect of crossbreeding on growth performance, meat quality, and production economics. A total of 451 unsexed day-old chicks were raised for 12 wk in a pure (Sasso X Sasso [**SS**]; Wassachie X Wassachie [**WW**]) and reciprocal cross (Sasso X Wassachie [**SW**]; Wassachie X Sasso [**WS**]) design. Data was collected on growth performance, meat quality, sensory evaluation, proximate analysis, and production economics. Genotype did not affect (*P* > 0.05) moisture, dry matter, ash, sensory evaluation, pH, and meat temperature. The carcass weights, final body weight, and cumulative weight gain of the hybrids were intermediate while the SS recorded the highest (*P* < 0.05) values. Drip loss between the WW and the reciprocal crosses was similar (*P* > 0.05) but lower (*P* < 0.05) than the SS genotype. Protein contents between the purebreds were similar likewise the crossbreds (*P* > 0.05). The SW cross recorded a higher (*P* < 0.05) lipid content compared to the WW cross while the WS recorded a higher (*P* < 0.05) protein content compared to the SS. The SS incurred higher feeding costs, and gross and net returns followed by the SW and then the WS with the WW having the lowest values. Crossbreeding improved growth performance, carcass traits, meat quality, chemical composition, and the gains in the crossbreds with the SW genotype having better results. The SW cross is recommended for better performance.

## INTRODUCTION

In the dynamics of poultry and livestock science, crossbreeding has gained substantial attention in Africa and around the globe for its potential to enhance various aspects of animals. It involves the mating of individuals from different genetic backgrounds to capitalize on the beneficial traits each possesses. Crossbreeding has been extensively explored to investigate its impact on growth performance (Mendonça et al., 2019), meat quality (Hoffman et al., 2003; Alonso et al., 2009), and the overall economics of production (Kahi et al., 2000).

Poultry meat and eggs rank among the most consumed animal-source foods globally (FAO, 2024). The demand is increasing due to population growth, urbanization, and improving incomes, particularly in developing countries (FAO, 2024). Its meat stands out as the preferred choice due to its affordability, low-fat content, and minimal cultural or religious restrictions (FAO, 2024). With high-quality protein and favorable fat profiles, chicken meat and eggs play a crucial role in human nutrition. In many developing countries, chicken production particularly indigenous chicken farming dominates mostly among the rural population (Boki, 2000; Sai'du et al., 2008) providing meat and eggs, which represent about 80% of total poultry stocks (Akinola and Essien, 2011). While commercial breeds of chicken have seen rapid growth in poultry consumption in Africa, the indigenous breeds remain significant in rural areas, even though faced with challenges of slow production rates due to poor genetic potential, they are prized for their distinctive taste and flavor (Guèye, 1998; Solomon et al., 2013).

Efforts have been made in the past to enhance the performance of indigenous using techniques like cross-breeding with commercial breeds and within-line selection. For instance, the cross between the local chicken with Marshal chicken resulted in crossbreds having higher body weights (Bassey et al., 2022), and meat yield also improved through indigenous-exotic breed cross (Martino et al., 2015). Other examples include the Potchefstroom Koekoek chicken of Ethiopia (Dessie and Getachew, 2016), the Boschveld Chicken of South Africa (Okoro *et al*., 2017), the Faso chicken breed of Burkina Faso (Agridigitale, 2022), and the Wassachiè chicken of Mali (Fomba, 2016) are products of crossbreeding local and exotic breeds, showcasing the potential for hybridization to meet diverse consumer preferences. In Togo, poultry production encompasses traditional and commercial operations, with small-scale farmers playing a significant role in domestic production. However, challenges such as limited finance access, technical expertise, and disease outbreaks impede sector expansion and efficiency. Consequently, Togo supplements domestic production with imports to address the supply-demand gap (Soviadan et al., 2021). During the period from 2021 to 2022, Togo's local meat production supplied 41,000 tonnes while imports of poultry meat reached 19,967 tonnes (Knoema, 2024).

The pursuit of optimized growth rates, meat quality, and production efficiency remains a central goal in the poultry industry, exploring the effects of crossbreeding on growth performance offers insights into how different genetic backgrounds interact and influence weight gain, feed conversion, and the overall efficiency of converting feed resources into meat. The quality of meat, a critical factor affecting consumer satisfaction and the competitive edge of the industry, is intricately connected to genetics (Castellini et al., 2002). Crossbreeding can influence meat characteristics like tenderness, marbling, and flavor (Pringle et al., 1997) subsequently shaping consumer preferences and market appeal. While scientific investigation delves into the growth and physiological aspects of poultry production, the economic dimension remains a crucial consideration. The economic viability of crossbreeding encompasses factors such as feed efficiency, production costs, and market prices. This evaluation offers stakeholders a comprehensive view of the potential benefits and challenges associated with adopting crossbreeding strategies in commercial production. This study was conducted to investigate the effect of crossbreeding on growth performance, meat quality, and production economics.

## MATERIALS AND METHODS

The study took place at the Poultry unit of the Regional Centre of Excellence for Avian Science (**CERSA**), situated at the University of Lome in Togo. Togo, positioned at latitude 6°7′55″N and longitude 1°13′22″E, experiences a tropical climate characterized by an average annual temperature of 26.6°C, an average annual precipitation of 1131 mm, and an annual average humidity of 90% (Google, 2023). March is noted as the warmest month, while August is considered the coolest month of the year (Google, 2023).

### Experimental Birds

A total of 451 unsexed day-old chicks hatched from the Hatchery Unit of CERSA were used for the experiment in a pure (Sasso X Sasso [**SS**]; Wassachie X Wassachie [**WW**]) and reciprocal cross (Sasso X Wassachie [**SW**]; Wassachie X Sasso [**WS**]) design. Each genotype was replicated thrice, they were reared on a deep litter system containing wood shavings as litter material for 12 wk and fed starter (21.20% crude protein; 2,926.40 kcal/kg metabolizable energy) and finisher (19.80% crude protein; 3,144.50 kcal/kg metabolizable energy) diets at the starter and finisher phases that lasted 6 wk each. Feed and water were made available at all times for consumption. An artificial lighting system using electric bulbs was provided during brooding while the rearing phase experienced approximately 12 h of natural daylight daily. All chicks were vaccinated following a program typical of the region. Care and management of the birds were similar at all times.

### Data Collection

Data was collected on growth performance, carcass, meat quality, sensory evaluation, proximate analysis, and production economics.

### Growth Performance

Data for growth performance was collected on cumulative feed intake, cumulative body weight gain, cumulative feed conversion ratio, and final body weight. Cumulative feed intake was measured as the total feed intake per bird. Cumulative body weight gain was measured as the total body weight gain per bird. Cumulative feed conversion was calculated as the total ratio of grams of feed intake to grams of total weight gain. Body weight was measured on the 12th wk of the experiment using a sensitive weighing scale calibrated in grams.

### Carcass Characteristics

Twenty birds from each genotype were starved for about 12 h before slaughter to obtain their dressing percentage and carcass cuts. The birds were euthanized by electrocution at 60 mA and slaughtered by cutting the knife through the jugular vein, bled, and scalded in hot water (60°C) for 55 s. The carcasses were passed through a machine and de-feathered mechanically. Standard carcass cuts and their weights were taken immediately and expressed as a percentage of live body weight.

### Proximate Analysis

The meat samples from the breasts of 18 different birds from each genotype were used for proximate analysis. Portions of meat were chopped into small pieces using a knife and grounded until a homogenous mixture was attained using a blender, and thereafter subjected to analysis for percentage moisture, dry matter, crude protein, fat, and total ash according to the AOAC (2005) technique. Dry matter content was determined by oven drying at 103°C until a stable weight was obtained; the Kjeldahal method was used for the analysis of total nitrogen content and crude protein content was calculated and expressed as a percentage. Total fat content was obtained by Soxhlet extraction using petroleum ether (boiling point [**BP**] 80–90°C); Ash content was determined by heating meat samples in a muffle furnace for 3 to 5 h at 600°C until a stable weight was obtained.

### Meat Quality

Meat samples collected from 18 breasts and thighs of the slaughtered chickens were analyzed for meat quality. Data on meat quality was collected on drip loss, pH, cooking loss, and sensory evaluation.

### Drip Loss

Drip loss was estimated at 24 and 72 h after meat storage following the method of Honikel (1998). The meat samples from the breast meat of known weights were suspended in polythene bags at 4°C in a refrigerator and weighed again after 24 and 72 h to determine their weight loss. Drip loss was calculated using the formula;

$$\text{Drip}\text{loss}\left(\%\right)=\left(W_{1}-W_{2}\right)/W_{1}\times 100$$

Where;

W1 = Initial weight of the sample

W2 = Final weight of the sample

### Cooking Loss

Cooking loss was assessed using the procedure outlined by Honikel (1998). Approximately 20g of meat samples from the breast were placed in a thin-walled plastic bag and immersed in a continuously boiling water bath (Memmert WNB 14), with the bag's opening extending above the water surface. The samples were cooked at a temperature of 95°C for 45 min to reach an internal temperature of 75°C. Upon reaching this point, the samples were removed from the water bath and cooled in running water for approximately 20 min to achieve thermal equilibrium. Subsequently, the meat was extracted from the bag, blotted dry, and weighed again to determine post-cooking weights. The cooking loss was then expressed as a percentage of the initial sample weight, as indicated below:

$$\text{Cooking}\text{loss}\left(\%\right)=\left(W_{1}-W_{2}\right)/W_{1}\times 100$$

Where;

W1 = Weight of the sample before cooking

W2 = Weight of the sample after cooking

### Sensory Evaluation

A 15-member taste panel actively participated in the evaluation of cooked meat samples obtained from the breast meat of 4 distinct genotypes (specifically, SS, WW, SW, WS). The composition of this panel was determined through random selection from a pool of potential consumers, all of whom were postgraduate students of the University of Lomé in Togo. This diverse panel consisted of 9 males and 6 females hailing from 6 different African countries: Togo, Cameroon, Benin Republic, Nigeria, Chad, and Burkina Faso. The average age of the panelists was 33 and ranged between 22 and 37 y. Before commencing the meat assessments, the panelists underwent 2 training sessions to familiarize themselves with the evaluation process. The meat samples for sensory evaluation were cooked using the procedure Honikel (1998) outlined. Approximately 20g of meat samples were placed in a thin-walled plastic bag and immersed in a continuously boiling water bath (Memmert WNB 14) at a temperature of 80°C without salt or seasoning, for about 45 min until a core temperature of 75°C was reached. The cooled meat was cut into identical cubes and served to the judges for evaluation. During 4 separate evaluation sessions, each panelist individually evaluated 5 essential sensory attributes of the meat, which included taste, flavor, tenderness, juiciness, and overall acceptability. They utilized the JAR acceptance test and rated 5 meat samples for each of these attributes on a 1 to 5 scale (Table 1), as described by Gacula et al*.* (2008). This resulted in each panelist delivering 4 assessments in total. Throughout the evaluation rounds, the meat samples from each genotype were presented twice, ensuring that each meat sample received a combined total of 5 assessments. This methodical approach guaranteed that each panelist conducted repetitive evaluations on a maximum of 20 meat samples spanning the various genotypes.

**Table 1:** Score sheet for Hedonic-scale rating

Sensory evaluation form using JAR acceptance test.					
Name: …………………………		Sex: …………………………		Date: …………………………	
Category: …………………………		Nationality…………………………		Age: …………………………	
Instruction:	Check one rating for each of the following; taste, flavor, tenderness, juiciness, and acceptability.				


Rating scale	Taste	Flavor	Tenderness	Juiciness	Acceptability
5	Very sweet	Very strong	Very tough	Very juicy	Extremely like
4	Sweet	Strong	Tough	Juicy	Like
3	Acceptable	Acceptable	Acceptable	Acceptable	Acceptable
2	Not sweet	Weak	Tender	Dry	Dislike
1	Tasteless	No flavor	Very tender	Very dry	Extremely dislike
Total					

### Temperature and pH Measurements

The temperature and pH of breast and thigh muscle samples were measured by adopting the procedure laid down by AOAC (2005) using a penetrating electrode of a portable pH meter (Food care HI99163). The probe was calibrated with pH 4 and 7 standard buffer solutions. Initial pH and temperatures were measured within 45 min after slaughter. Subsequent readings were taken only on the breast at 24 and 72 h postmortem.

### Economics of Production

The economics of production was analyzed using budget analysis and profitability ratios as stipulated by Adeoti and Olawumi (2013). The analysis involves the deduction of the total variable costs (in USD) from the total revenue of the live weight (in USD) to obtain the gross margin from each genotype. The total variable cost of the production includes the cost of day-old chicks, labor, feed, veterinary services, medication, and other miscellaneous services.

(1) Grossmargin=grossrevenue−totalvariablecost

It is given by the formula:

(2) $$GM=\sum_{i=1}^{n}\text{Pi}\;\text{Yi}-\text{Ci}$$

Where;

GM = Gross margin

Pi = Farm gate price per kg of meat of i^th^ genotype

Yi = Total live weight in kg of meat of i^th^ genotype

Ci = Total variable cost incurred on the genotype n = Total no of birds per i^th^ genotype

The profitability ratios include the benefit cost ratio (**BCR**), profitability index (**PI**), and the rate of return on investment (**ROI**). Because the fixed costs were equal across genotypes, they were excluded from the analysis, and only the gross margin was utilized as a proxy for net profit. The economic analysis was done by evaluating the following parameters: total feed consumed/bird/genetic group, the total cost of feeding/bird/genetic group, feed cost per kg body weight, and gross revenue/bird (the birds were sold at 1,000/kg live weight).

(3) Benefit−costratio=TR/TC

(4) Profitablyindex=NP/TR=GM/TR

(5) Rateofreturnoninvestment=NP/TC×100=GM/TC×100

Where;

TR= Total revenue (value of the total live weight of the genotype)

TC = Total cost of production

NP = Net profit

To determine the average productivity of the imputes used for production 4 indicators were assessed: feed, veterinary services, labor, and cost productivities, and were estimated as follows;

*Feed productivity*

(6) $$\text{Feed}\;\text{productivity}=\sum_{i=1}^{n}\text{Yi}\;/Qf$$

Where Qf = Quantity of feed in kg

*Veterinary services productivity*

(7) $$\text{Veterinary}\;\text{services}=\sum_{i=1}^{n}\text{Yi}\;/Qv$$

Where Qv = Cost of veterinary services in USD

*Labor productivity*

(8) $$\text{Labor}\;\text{productivity}\;=\sum_{i=1}^{n}\text{Yi}\;/Ql$$

Where Ql = Quantity of labor in man days

*Cost productivity*

(9) $$\text{Cost}\;\text{productivity}=\sum_{i=1}^{n}\text{Yi}\;/TC$$

Where TC = Total cost of production in USD

### Data Analysis

Minitab (Version 19) and R statistical software (Version 4.2.2) packages were used for data analyses. All data obtained as proportions were transformed on R statistics software before analysis, using the square root arcsine method as specified by Sokal and Rohlf (1995). For this transformation, each percentage value (**X**) was subjected to the following formula:

$$Y=\text{arcsin}\sqrt{\left(X/100\right)}$$

Where Y represents the transformed value corresponding to each percentage value X.

The effects of genotype on the parameters measured were analyzed using ANOVA on Minitab, and the differences in means were tested for significance (*P* < 0.05) using Tukey's test. The following linear model was used for data analysis:

$$Y_{\text{ij}}=\mu +\text{Gi}+e_{\text{ij}}$$

Where:

Y_ij_ = The observation

µ = Overall mean

G_i_ = Effect of the i^th^ genotype (i= SS, SW, WS, WW) e_ij_ = Random error

## RESULTS

As indicated in Table 2, there was no significant effect (*P* > 0.05) of genotype on FCR, while other parameters, including CFI, CBWG, and FBW, were statistically different (*P* < 0.05). The pure Sasso and the reciprocal crosses consumed similar (*P* > 0.05) amounts of feed which were higher (*P* < 0.05) than the feed intake of the pure Wassachie cross. The final body weight and cumulative weight gain were higher (*P* < 0.05) in the pure Sasso genotype, followed by the reciprocal crosses with the pure Wassachie cross showing the lowest values.

**Table 2:** Effect of genotype on cumulative growth parameters of the pure and reciprocal crosses.

Parameter	SS	SW	WS	WW	*P*-value
CFI (g)	4228.41 ± 233^a^	3461.86 ± 131^a^	3344.71 ± 260^a^	2375.99 ± 188^b^	0.001
CBWG (g)	1605 ± 103^a^	1220.20 ± 17.20^b^	982.00 ± 38.80^b^	702.9 ± 44.10^c^	0.000
CFCR	2.64 ± 0.03	2.84 ± 0.11	3.43 ± 0.37	3.89 ± 0.04	0.059
FBW (g)	1643 ± 105^a^	1252.90 ± 17.10^b^	1020.20 ± 38.70^b^	734.6 ± 44.60^c^	0.000

Means ± SEM with different superscripts.

abc Across rows differ significantly (*P* < 0.05), SS, ♂ Sasso x ♀ Sasso; SW, ♂ Sasso x ♀ Wassachie; WS, ♂Wassachie x ♀ Sasso; WW, ♂ Wassachie x ♀ Wassachie; CFCR, cumulative feed conversion ratio; FBW, final body weight; CBWG, cumulative body weight gain; CFI, cumulative feed intake. (SS = 110; WW = 113; SW = 113; WS = 115)

From Table 3, the weights of carcasses and carcass portions of the pure Sasso were significantly (*P* < 0.05) heavier than those of the pure Wassachie. The SS and SW genotypes had similar (*P* > 0.05) weights in all the parameters measured except for the breast weight which was heavier (*P* < 0.05) in the SS cross, likewise, the WS and WW genotypes reported similar (*P* > 0.05) carcasses and carcass portions except for heavier breast weight (*P* < 0.05) in the WS genotype. The carcasses and carcass portions between the crossbreds were similar (*P* > 0.05) when measured. The weights of the thigh from the reciprocal crosses were similar (*P* > 0.05) to that of the pure Sasso cross.

**Table 3:** Effect of genotype on carcass and carcass portions of the pure and reciprocal crosses (G).

Parameter	SS	SW	WS	WW	*P-*value
Fasted Wt	1381.80 ± 11^a^	1228.40 ± 40.70^ab^	1027.10 ± 32.80^bc^	803.90 ± 22.50^c^	0.001
Slt Wt	1331.20 ± 11^a^	1170.10 ± 36.90^ab^	953.30 ± 24.80^bc^	737.50 ± 39.90^c^	0.001
Def Wt	1194.70 ± 10^a^	1046.00 ± 38.40^ab^	863.50 ± 36.70^bc^	670.90 ± 21.30^c^	0.001
Carcass Wt	861.20 ± 79.80^a^	755.10 ± 28.5^ab^	637.10 ± 59.90^ab^	531.30 ± 10.80^b^	0.010
Breast Wt	262.00 ± 16.50^a^	204.53 ± 4.53^b^	168.80 ± 5.47^b^	109.94 ± 3.22^c^	0.000
Thigh	69.70 ± 7.18^a^	65.10 ± 3.90^a^	53.22 ± 2.02^ab^	41.80 ± 2.46^b^	0.008
Drum stk	69.90 ± 6.79^a^	62.26 ± 2.65^ab^	49.79 ± 1.85^bc^	40.43 ± 1.69^c^	0.003
Breast yld	167.90 ± 18.20^a^	143.53 ± 2.62^ab^	116.49 ± 4.22^b^	91.07 ± 2.50^c^	0.002

Means ± SEM with different superscripts

abc Across rows differ significantly (*P* < 0.05), SS, ♂ Sasso x ♀ Sasso; SW, ♂ Sasso x ♀ Wassachie; WS, ♂Wassachie x ♀ Sasso; WW, ♂ Wassachie e x♀ Wassachie; Fasted Wt- fasted weight; Slt Wt, slaughtered weight; Def Wt, defeathered weight; Carcass Wt, carcass weight; Breast Wt, breast weight; Drum stk, drum stick; Breast yld, breast yield; N = 20.

The effects of genotype on moisture loss, temperature pH, and proximate composition of the pure and reciprocal crosses genotype are shown in Table 4. Genotype did not show any significant effect (*P* > 0.05) on cooking loss. The percentage drip loss (24 and 72) of the pure Wassachie and the reciprocal crosses was similar (*P* > 0.05) but lower (*P* < 0.05) compared to the pure Sasso, except between the SW and SS crosses which was similar (*P* < 0.05). There was no significant effect (*P* > 0.05) of genotype on the pH and temperature of the meat at all times. There was no significant (*P* > 0.0) effect of genotype on moisture, dry matter, and ash contents. The protein and lipid contents from the meat of the purebreds were similar (*P* > 0.05), likewise the reciprocal crosses. Crossbreeding induced a higher (*P* < 0.05) lipid content in the SW cross compared to the WW cross and a higher (*P* < 0.05) protein content in the WS cross compared to the SS. The values for proximate analysis between the purebreds did not differ (*P* > 0.05).

**Table 4:** Effect of genotype on moisture loss, temperature and ph and proximate composition of the pure and reciprocal crosses.

Parameter	SS	SW	WS	WW	*P*-value
Water Loss (%)					
Drip loss _24_	6.50 ± 0.57^a^	4.14 ± 0.58^b^	4.35 ± 0.52^ab^	3.97 ± 0.13^b^	0.020
Drip loss _72_	8.85 ± 0.52^a^	6.33 ± 0.24^b^	7.22 ± 0.51^ab^	6.71 ± 0.22^b^	0.010
Cooking loss	23.53 ± 2.50	23.78 ± 1.09	26.58 ± 1.88	25.79 ± 2.35	0.664
Temperature (°C) and pH					
pH _0_ breast	5.82 ± 0.04	5.86 ± 0.15	5.81 ± 0.02	5.91 ± 0.20	0.2162
pH _24_ breast	5.78 ± 0.03	5.73 ± 0.07	5.75 ± 0.03	5.80 ± 0.03	0.3162
pH _72_ breast	5.54 ± 0.06	5.71 ± 0.44	5.62 ± 0.11	5.63 ± 0.34	0.319
Tmp _0_ breast	30.30 ± 0.18	29.74 ± 0.13	30.00 ± 0.30	28.29 ± 1.51	0.333
Tmp _24_ breast	20.48 ± 0.41	21.04 ± 0.36	22.33 ± 1.00	21.76 ± 0.39	0.224
Tmp _72_ breast	20.12 ± 0.16	20.71 ± 0.71	21.71 ± 0.22	21.05 ± 0.76	0.277
pH _0_ Thigh	6.09 ± 0.02	6.00 ± 0.00	6.09 ± 0.05	5.96 ± 0.04	0.051
T_0_ Thigh	29.63 ± 0.11	29.65 ± 0.12	29.39 ± 0.07	28.14 ± 1.57	0.518
Proximate composition (%)					
Moisture	69.87 ± 0.77	67.56 ± 1.62	70.38 ± 0.43	71.96 ± 1.06	0.100
Dry matter	30.14 ± 0.77	32.44 ± 1.62	29.63 ± 0.43	28.04 ± 1.06	0.100
Protein	78.41 ± 0.26^b^	79.43 ± 0.68^ab^	80.29 ± 0.14^a^	79.14 ± 0.19^ab^	0.001
Lipid	6.27 ± 0.36^ab^	9.89 ± 0.98^a^	4.57 ± 1.56^b^	5.37 ± 0.25^b^	0.009
Ash	4.46 ± 0.41	4.01 ± 0.77	3.81 ± 0.10	4.10 ± 0.15	0.340

Means ± SEM with different superscripts.

ab Across rows differ significantly (*P* < 0.05), SS, ♂ Sasso x ♀ Sasso; SW, ♂ Sasso x ♀ Wassachie; WS, ♂Wassachie x ♀ Sasso; WW, ♂ Wassachie x ♀ Wassachie; Drip loss 24 , drip loss as 24 h; Drip loss 72 , drip loss at 72 h; N = 20. Tmp 0, temperature at 0 h; Tmp 24, temperature at 24 h; Tmp 72, temperature at 72 h; pH 0, pH at 0 h; pH 24, pH at 24 h; pH 72, pH at 72 h; N = 20

The effect of genotype on the sensory evaluation of the meat is presented in Table 5. Genotype did not have any significant (*P* > 0.05) effect on the sensory evaluation of the meat.

**Table 5:** Effect of genotype on sensory evaluation of the pure and reciprocal crosses.

Parameter	SS	SW	WS	WW	*P*-value
Taste	3.81 ± 0.11	3.76 ± 0.11	3.71 ± 0.11	3.79 ± 0.15	0.947
Flavor	3.47 ± 0.17	3.17 ± 0.19	3.21 ± 0.15	3.40 ± 0.19	0.572
Tenderness	2.69 ± 0.16	2.91 ± 0.22	2.90 ± 0.18	2.80 ± 0.20	0.817
Juiciness	3.47 ± 0.11	3.57 ± 0.17	3.46 ± 0.15	3.68 ± 0.15	0.690
Acceptability	3.67 ± 0.10	3.86 ± 0.11	3.64 ± 0.10	3.73 ± 0.13	0.524

SS, ♂ Sasso x ♀ Sasso; SW, ♂ Sasso x ♀ Wassachie; WS, ♂Wassachie x ♀ Sasso; WW, ♂ Washashe x ♀ Wassachie; N = 15.

The economics of production of the genotypes and their reciprocal genotype crosses is indicated in Table 6. The cost incurred in feeding, gross, and net returns were higher in the pure Sasso cross followed by the SW and then the WS with the WW showing the lowest values.

**Table 6:** Economics of production of the pure and reciprocal crosses (USD).

Parameter	SS	SW	WS	WW
Feed cost/kg	0.59	0.59	0.59	0.59
Total FI/b (kg)	4.27	3.50	3.40	2.40
Total feed cost	2.52	2.07	2.00	1.42
*Live chicken*				
Price/kg	2.54	2.54	2.54	2.54
Gross returns (Total)	4.17	3.18	2.59	1.86
Net returns on LV	1.65	1.11	0.59	0.44

SS, ♂ Sasso x ♀ Sasso; SW, ♂ Sasso x ♀ Wassachie; WS, ♂Wassachie x ♀ Sasso; WW, ♂ Wassachie x ♀ Wassachie; Total FI/b , total feed intake per bird; Net returns on LV, net returns.

## DISCUSSION

The interplay between genetics and economics is important in understanding how crossbreeding can be harnessed to optimize sustainable poultry production, meat quality, and economic viability. This study sheds light on the multifaceted ways in which the strategic fusion of genetics in chicken farming influences economic viability and the culinary appeal of the end product, offering insights into a nuanced and promising dimension of modern poultry husbandry.

Capitalizing on the complementary strengths of different breeds of chicken while mitigating potential weaknesses can enhance growth performance traits and carcasses. A higher (*P* < 0.05) BWG was recorded in the pure Sasso genotype compared to the pure Wassachie genotype which translated to a higher body weight suggesting a faster growth rate due to the differences in genotype. The body weights and BWGs of the reciprocal crosses were similar (*P* > 0.05) and intermediate to the pure breeds implying an improvement in these traits influenced by crossbreeding. Total feed consumed by the pure Sasso was significantly (*P* < 0.05) higher compared to the pure Wassachie which is expected of birds having a higher body weight, Khawaja et al. (2012) also reported a higher body weight and weight gain in the exotic breed. Crossbreeding induced a similar (*P* > 0.05) total feed intake between the reciprocal crosses and pure Sasso which was higher (*P* < 0.05) than the total feed consumed by the pure Wassachie cross implying the influence of the Sasso genes. This was expected as birds with higher body weights would consume more feed (Nwachukwu et al. 2006). The results agree with the report of Khawaja et al. (2012) who also reported an improvement in growth traits of the crossbreds when Rhode Island red was crossed with Fayoumi chicken

The carcasses and carcass portions of the pure Sasso genotype were heavier compared to the pure Wassachie genotype which indicates the superiority of the pure Sasso genotype. This superiority was evident among the crossbred chickens which also recorded higher carcass and carcass portions compared to the WW genotype, primarily driven by the dominance effect that reached statistical significance exclusively within the SW genotype. Nevertheless, the breast weights showed no significant differences (*P* > 0.05) observed between the crossbred chickens and the pure Sasso breed, which also produced similar breast yields (*P* > 0.05). Tabinda et al. (2012) and Keambou et al. (2015) also reported an improvement in the carcasses of the crossbreds when the local chickens and exotic breeds were crossed.

To evaluate the water-binding characteristics of the meat samples from the different genotypes, water loss was measured. The water that meat releases without any external force, except for capillary forces is commonly referred to as drip (Mir et al., 2017). Because oxygen supply is unavailable post-mortem, it leads to the production of lactic acid. This results in a pH decline that causes protein denaturation and subsequently leads to a reduction in water-holding capacity (Mir et al., 2017). The pH and temperature assessment at various intervals showed no effect of genotype (*P* > 0.05) this agrees with the results of Fernandez et al. (2001) who also reported similar pH declines in pure and crossbred turkeys. However, a higher drip loss at 24 and 72 h was recorded in the pure Sasso breed compared to the pure Wassachie cross (*P* < 0.05). On the contrary, the report of Fernandez et al. (2001) showed a higher drip loss in the slow-growing line when fast and slow-growing turkeys were crossed. Berri et al. (2001) in an experiment using 4 broiler genotypes reported a similar drip loss but a slower pH decline in the selected line. The difference (*P* < 0.05) in drip loss despite similar pH and pH decline could be due to differences in the muscle structure of the genotypes. According to Huo et al. (2021), variations exist in muscle fiber characteristics among breeds, these variations are responsible for the differences in meat quality both across and within breeds. Although the crossbred chickens in this study showed comparable drip rates (*P* > 0.05), the SW genotype exhibited lower drip loss compared to the pure Sasso, which displayed drip loss rates similar to those of the WW genotype (*P* > 0.05). This variability could be attributed to the maternal influence of the Wassachie chicken.

The result of the proximate analysis of meat samples between the purebreds showed no significant (*P* > 0.05) effect of genotype, this agrees with the report of Khawaja et al. (2012). On the other hand, Van Marle-Ko¨ster and Webb (2000) reported an effect of strain on the chemical composition of meat in a study involving 6 commercial broiler strains. Crossbreeding induced a higher (*P* > 0.05) protein content in the WS cross compared to the SS genotype due to the inheritance of the Wassachie genes, and a higher (*P* > 0.05) lipid content in the SW in comparison to the WW and WS genotypes (*P* > 0.05), indicating the effect of sex chromosomes. The report of Khawaja et al. (2012) showed no significant difference in the proximate composition of the carcasses between the purebreds and reciprocal crosses. According to Castellini et al., (2002), chickens exhibited different degrees of meat maturity, protein, and moisture content at the same age as the Ross, Kabir, and Robusta maculate chickens were studied. This suggests that the chemical composition of meat may vary depending on the genotype.

The quality of chicken meat directly influences consumer preferences and the marketability of poultry products as meat with superior properties will command higher demand. The result for sensory evaluation showed no significant (*P* > 0.05) effect of genotype on all parameters measured. The pure Sasso genotype recorded a higher (*P* > 0.05) score for taste and flavor compared to the pure Wassachie genotype. The majority of the panelists preferred the taste and flavor of the WW genotype to the crossbreds. However, the WW genotype meat appeared tenderer and juicier than that of the pure Sasso genotype. Differences in tenderness among meat samples could be related to differences in the chemical or physical properties of meat (Smith et al., 1973; Smith and Carpenter, 1974). The Wassachie genotype recorded higher moisture (*P* > 0.05) which could have accounted for the tenderer meat. There's a relationship between tenderness and juiciness. The more tender the meat, the more quickly the juices are released by chewing, and the juicier the meat appears (Smith and Carpenter, 1974). Crossbreeding induced a higher (*P* > 0.05) tenderness in the crossbreds due to the inheritance of the Wassachie genes. The overall acceptability score of the Wassachie was higher (*P* > 0.05) than the pure Sasso indicating its preference which is expected as meat from the indigenous chicken is preferred in Africa (Guèye, 1998). In comparison, the SW genotype recorded the highest acceptability score (*P* > 0.05) which implies a maternal effect of the Wassachie breed.

The economics of production for the pure and reciprocal crosses reported the highest feed cost incurred in the pure Sasso cross this is due to the higher feed consumed comparably (Table 6). Higher returns per gram of body weight are a result of higher body gains recorded in the pure Sasso genotype (Table 6). The same explanation could be said for comparing the reciprocal crosses and the WW cross. Between the reciprocal crosses, the SW cross had higher values for gross and net returns implying higher profitability.

## CONCLUSIONS

The pure Sasso genotype demonstrated better productivity, higher costs in feeding, gross and net returns in comparison to the reciprocal crosses, with the WW genotype indicating the lowest values. Crossbreeding improved the growth performance, carcass traits, meat quality, and chemical composition of the meat, it also improved the gains of the crossbreds. Between the reciprocal crosses, the SW genotype produced a better performance. Thus, considering production costs, market demand, and various performance parameters, the use of the SW cross is recommended for use for better results.

## DISCLOSURES

The authors declare no conflicts of interest.

## References

[bib0001] Adeoti A., Olawumi S. (2013). Economic assessment of raising different broiler strains. Asian J. Poult. Sci..

[bib0002] Agridigitale. 2022. Faso, Goliath, Wassachiè chicken: what's the difference?https://agridigitale.net/article/le_poulet_faso_goliath_wassachi_quelle_diffrence. Accessed Apr. 8, 2024.

[bib0003] Akinola L.A.F., Essien A. (2011). Relevance of rural poultry production in developing countries with special reference to. Africa. World's Poultry Sci. J..

[bib0004] Alonso V., del Mar Campo M., Español S., Roncalés P., Beltrán J.A. (2009). Effect of crossbreeding and gender on meat quality and fatty acid composition in pork. Meat Sci.

[bib0005] AOAC (2005).

[bib0006] Bassey O.A., Akpan U., Ikeobi C.O.N., Adebambo O.A., Idowu O.M.O., Ilori O.J. (2022). Performance of Nigerian indigenous chicken genotypes and their crosses with Marshal breed. AUJST.

[bib0007] Berri C., Wacrenier N., Millet M., Bihan-duval E.L.e (2001). Effect of selection for improved body composition on muscle and meat characteristics of broilers from experimental and commercial lines. Poult. Sci.

[bib0008] Boki K.J. (2000). Poultry industry in Tanzania with emphasis on small-scale rural poultry. Proceeding of workshop, Morogoro, Tanzania, 22-25th May, 2000.

[bib0009] Castellini C., Mugnai C., Bosco A.D. (2002). Meat quality of three chicken genotypes reared according to the organic system. Italian J. Food Sci.

[bib0010] Dessie T. and Getachew F., The Potchefstroom Koekoek Breed. African Chicken Genetic Gains Factsheet 3, 2016, ILRI; Nairobi, Kenya https://hdl.handle.net/10568/72964. Accessed Jan. 10, 2024.

[bib0011] FAO. 2024. Gateway to poultry production and products.https://www.fao.org/poultry-production-products/socio-economic-aspects/poultry-chain/en/. Accessed Apr. 7, 2024.

[bib0012] Fernandez X., Sante V., Baeza E., Lebihan-Duval E., Berri C., Remignon H., Babile R., Pottier G.L.e, Millet N., Berge P., Astruc T. (2001). Post mortem muscle metabolism and meat quality in three genetic types of turkey. Br. Poult. Sci..

[bib0013] Fomba H. (2016). Genetic improvement of poultry: The performance of “Wassa Chè” chicken. JSTM.

[bib0014] Gacula M., Mohan P., Faller J., Pollack L., Moskowitz H.R. (2008). Questionnaire practice: What happens when the JAR scale is placed between two “overall” acceptance scales?. J. Sens. Stud..

[bib0015] Google. 2023. Satellite View and Map of the City of Lome, Togo.https://www.nationsonline.org/oneworld/map/google_map_Lome.htm. Accessed Mar. 22, 2023.

[bib0016] Guèye E.F. (1998). Village egg and fowl meat production in. Africa. World's Poult. Sci. J..

[bib0017] Hoffman L.C., Muller M., Cloete S.W.P., Schmidt D. (2003). Comparison of six crossbred lamb types: sensory, physical and nutritional meat quality characteristics. Meat Sci.

[bib0018] Honikel K.O. (1998). Reference methods for the assessment of physical characteristics of meat. Meat Sci.

[bib0019] Huo W., Weng K., Gu T., Zhang Y., Zhang Y., Chen G., Xu Q. (2021). Effect of muscle fiber characteristics on meat quality in fast- and slow-growing ducks. Poult. Sci..

[bib0020] Kahi, A. K., W. Thorpe, G. Nitter, J. A. M. Van Arendonk, and C. F. Gall. 2000. Economic evaluation of crossbreeding for dairy production in a pasture-based production system in Kenya. 65: 167–184.

[bib0021] Keambou T.C., Mboumba S., Touko B.A.H., Bembide C., Mezui T.M., Tedongmo A.M.Y., Manjeli Y. (2015). Growth performances, carcass and egg characteristics of the local chicken and its first-generation reciprocal crossbreds with an exotic strain in Cameroon. Adv. Anim. Vet. Sci..

[bib0022] Khawaja T., H.Khan S., Mukhtar N., Ali M.A., Ahmed T., Ghafar A. (2012). Comparative study of growth performance, egg production, egg characteristics and haemato-biochemical parameters of Desi, Fayoumi and Rhode Island Red chicken. J Appl Anim Res.

[bib0023] Knoema. 2024. Poultry meat imports.https://knoema.com/atlas/Togo/topics/Agriculture/Trade-Import-Quantity/Poultry-meat-imports-quantity. Accessed Feb. 2024.

[bib37] Martino C., Massimo D.M., Mauro P., Chiara R. (2015). Carcass characteristics and meat quality traits of the padovana chicken breed, a commercial line, and their cross. Ital. J. Anim. Sci..

[bib0024] Mendonça F.S., MacNeil M.D., Leal W.S., Azambuja R.C.C., Rodrigues P.F., Cardoso F.F. (2019). Crossbreeding effects on growth and efficiency in beef cow-calf systems: evaluation of Angus, Caracu, Hereford and Nelore breed direct, maternal and heterosis effects. Transl Anim Sci.

[bib0025] Mir N.A., Rafiq A., Kumar F., Singh V., Shukla V. (2017). Determinants of broiler chicken meat quality and factors affecting them: a review. J. Food Sci. Technol.

[bib0026] Nwachukwu E.N., Ibe S.N., Ejekwu K., Oke U.K. (2006). Evaluation of growth parameters of main and reciprocal crossbred normal, naked and frizzle chickens in a humid tropical environment. J. Anim. Vet. Adv.

[bib0027] Okoro V.M.O., Ravhuhali K.E., Mapholi I.T.H., Mbajiorgu E.F., Mbajiorgu C.A. (2017). Effect of age on production characteristics of Boschveld indigenous chickens of South Africa reared intensively. S. Afr. J. Anim. sci..

[bib0028] Pringle T.D., Williams S.E., Lamb B.S., Johnson D.D., West R.L. (1997). Carcass characteristics, the calpain proteinase system, and aged tenderness of Angus and Brahman crossbred steers. J. Anim. Sci..

[bib0029] Sai'du L., Wakama A.M., Waziri I.M., Abdu P.A. (2008). Pages 696–698 in Proceeding of the 13th Annual Conference of the Animal Science Association of Nigeria (ASAN).

[bib0030] Smith G.C., King G.T., Carpenter Z.L. (1973). Laboratory Exercises in Elementary. Meat Science.

[bib0031] Smith G.C., Carpenter Z.L. (1974). Pages 147–83 in Fat Content and Composition of Animal Products: Proceedings of a Symposium Washington, DC.

[bib0032] Sokal R.R., Rohlf F.J. (1995).

[bib0033] Solomon Z., Kassa B., Agza B., Alemu F. (2013).

[bib0034] Soviadan M.K., Enete A.A., Okoye C.U., Dossa K.F. (2021). Extensive and improved traditional poultry farming in togo: a comparative analysis of socioeconomic characteristics of farmers. ESJ.

[bib0035] Tabinda K., Sohail H.K., Nasir M., Abida P. (2012). Comparative study of growth performance, meat quality and haematological parameters of Fayoumi, Rhode Island Red and their reciprocal crossbred chickens. Ital. J. Anim. Sc..

[bib0036] Van Marle-Ko¨ster E., Webb E.C. (2000). Carcass characteristics of South African native chicken lines. Afr. J. Anim. Sci..

